# Supplementary material for: Perceived Determinants of Children’s Inadequate Sleep Health. A Concept Mapping Study among Professionals
Source: Int J Environ Res Public Health. 2020 Oct 7;17(19):7315. doi: 10.3390/ijerph17197315 (PMC7578965; doi:10.3390/ijerph17197315)
Supplement: Supplementary file 1 [file ijerph-17-07315-s001.pdf]

Supplementary A

Table A1. Characteristics of "inadequate sleep" according to professionals.

	**Defined as "inadequate sleep" by professionals**
**Always[a]**	Problems sleeping through the night, Problems falling asleep, Negative effects on daytime functioning, Not waking up rested
**Often[b]**	Inadequate sleep duration, Waking up (too) early, Going to bed too late, Restless sleeping
**Sometimes[c]**	Not being able to sleep alone, Resisting going to sleep, Oversleeping, Experiencing nightmares
**Rarely[d]**	Changing bedtimes, Sleep walking, Bed wetting, Demanding certain activities, Snoring, Getting up during the night, Having trouble getting up, Behaviours at night, Night terrors, Breathing problems, Teeth grinding, Painful legs at night, Insufficient sleep quality, Head banging, Multiple (≥ 3) nights of inadequate sleep, Negative effects on the parents and the family , Screen use before sleeping, Parents who do not set rules

[a] Definition was mentioned frequently by all groups; [b] Definition was mentioned frequently by two groups. [c] Definition was mentioned less frequently by all groups; [d] Definition was mentioned by one group.

Supplementary B

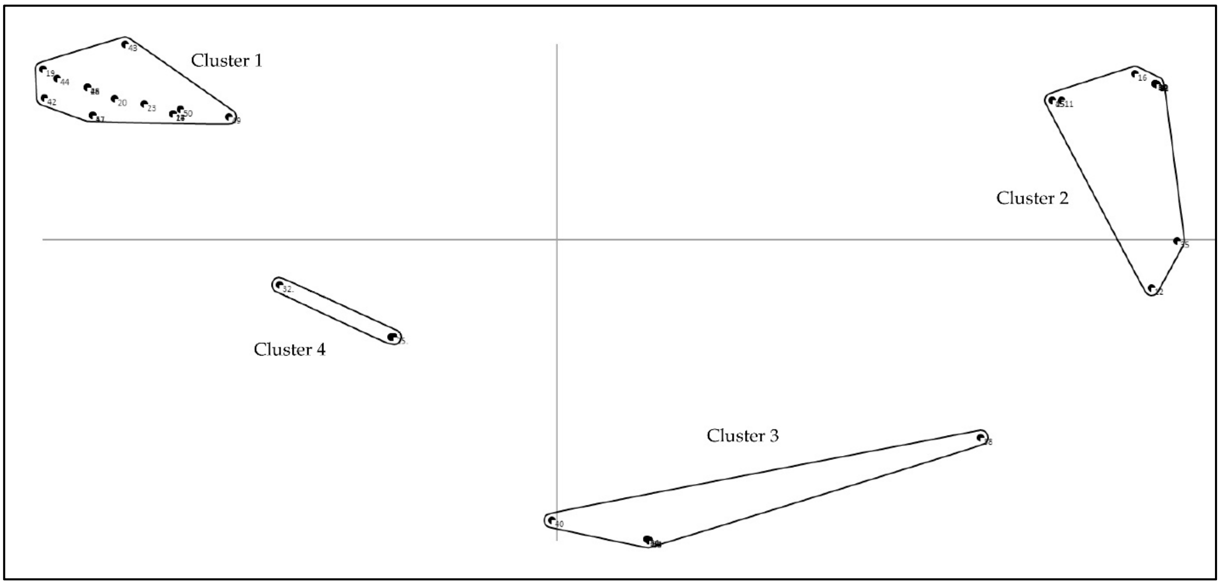

Figure A1. Concept map doctors. In this map, each point reflects one idea. Ideas that were grouped together more often appear closer to each other on the map. Ideas never/rarely grouped together appear widely separated on the map. Clusters are groups of ideas that were grouped together most often and reflect ideas that are conceptually related according to the participants in this group. The defined cluster names in this concept maps were: Cluster 1: Pedagogical-environmental factors (e.g. screen use before bedtime, inadequate daytime physical activity, inconsistent sleep schedule); Cluster 2: Psychosocial factors (e.g. worrying, fear, stressful family situation); Cluster 3: Medical factors (e.g. physical complaint, medical complaint); Cluster 4: Sleep-environmental factors (e.g. not the right temperature, bedroom sharing).

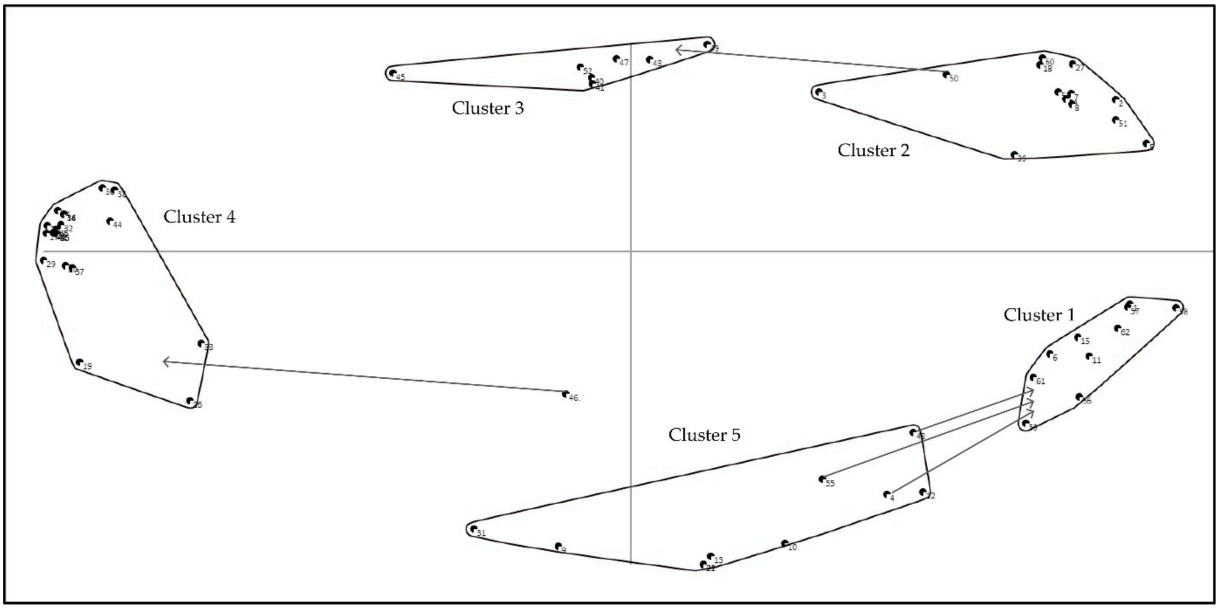

Figure A2. Concept map nurses. In this map, each point reflects one idea. Ideas that were grouped together more often appear closer to each other on the map. Ideas never/rarely grouped together appear widely separated on the map. Clusters are groups of ideas that were grouped together most often and reflect ideas that are conceptually related according to the participants in this group. The defined cluster names in this concept maps were: Cluster 1: Pedagogical-environmental factors (e.g. inadequate structure of the day, bedtime procrastination, inconsistent sleep schedule); Cluster 2: Daytime and evening activities (e.g. evening physical activity, screen use before bedtime, inadequate time to relax); Cluster 3: Medical factors (e.g. illness, medical complaint); Cluster 4: Psychosocial factors (e.g. fear, worrying, stressful family situation); Cluster 5: Sleep-environmental factors (e.g. noise outside, too much light). Arrows indicate an idea is reallocated by researchers; idea 4 'Secretly reading a book in bed' was moved from Cluster 5 to Cluster 1, idea 46 'The child's temperament' was moved to Cluster 4, idea 48 'Refusing to go to bed' was moved from Cluster 5 to Cluster 1, idea 50 'Having a disturbed biological clock' was moved from Cluster 2 to Cluster 3, and idea 55 'Comparing oneself to classmates who go to bed later' was moved from Cluster 5 to Cluster 1.

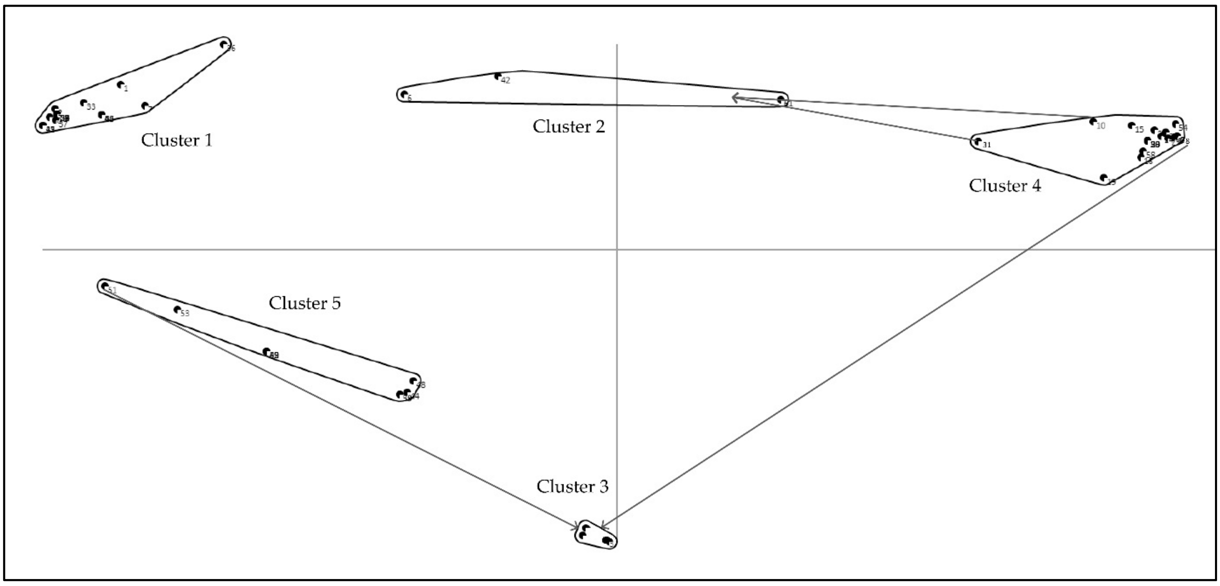

Figure A3. Concept map sleep experts. In this map, each point reflects one idea. Ideas that were grouped together more often appear closer to each other on the map. Ideas never/rarely grouped together appear widely separated on the map. Clusters are groups of ideas that were grouped together most often and reflect ideas that are conceptually related according to the participants in this group. The defined cluster names in this concept maps were: Cluster 1: Sleep hygiene factors (e.g. inadequate bedtime, inconsistent sleep schedule, no bedtime

routine); Cluster 2: Pedagogical-environmental factors (e.g. bedtime procrastination, lack of clear rules); Cluster 3: Medical factors (e.g. physical complaint, medical complaint, sleep disorder); Cluster 4: Psychosocial factors (e.g. worrying, fear, stressful family situation); Cluster 5: Sleep-environmental factors (e.g. noise outside, not the right temperature, bedroom sharing). Arrows indicate an idea is reallocated by researchers; idea 8 'Mental problems' was moved from Cluster 4 to Cluster 3, idea 10 'Refusing to listen and continuing to test the limits when going to bed' was moved from Cluster 4 to Cluster 2, idea 31 'Parents who do not set clear rules or who are too strict' was moved from Cluster 4 to Cluster 2, idea 51 'Medication use with effects on sleep, taking medication at the wrong time' was moved from Cluster 5 to Cluster 3.

Supplementary C

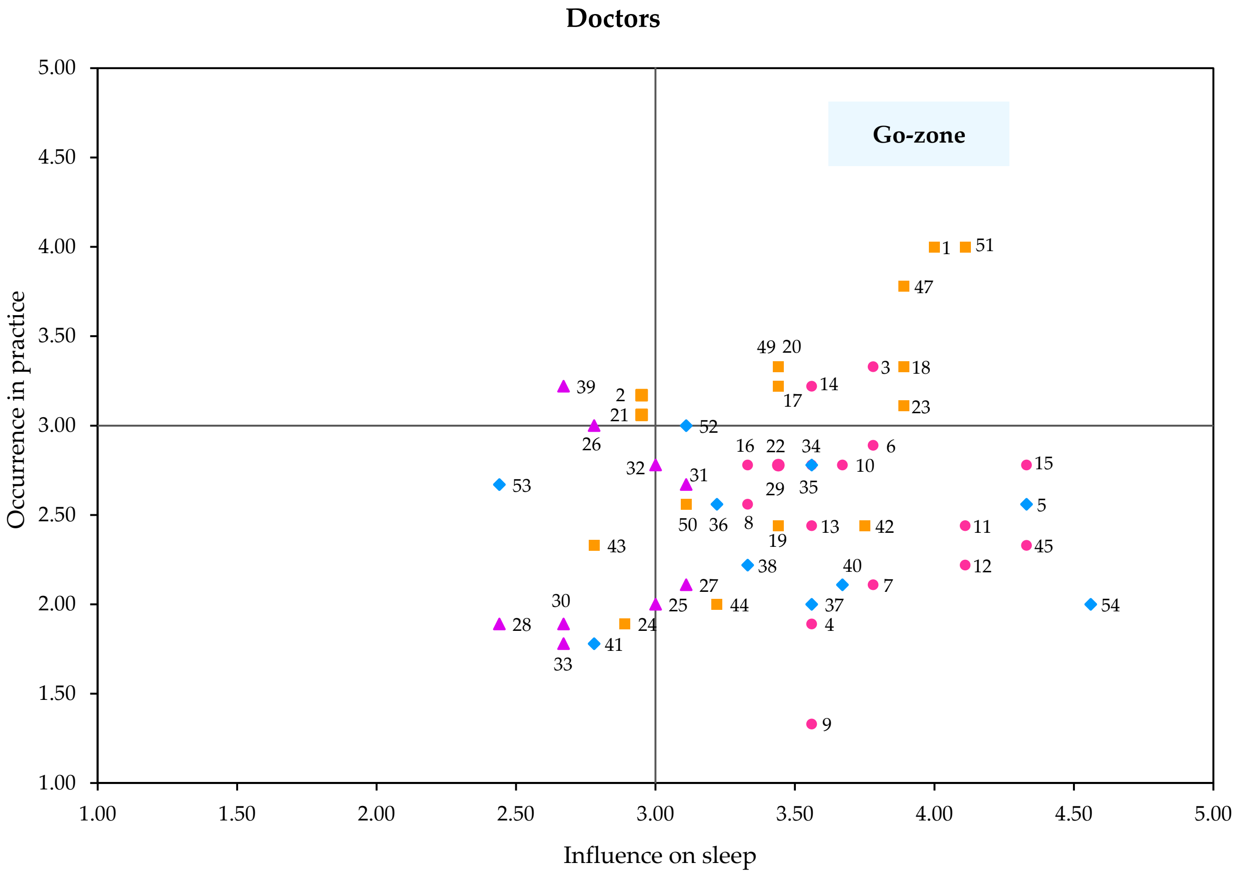

Figure A4. Go-zone plot doctors. Coloured shapes indicate to what cluster this idea belongs: ■ pedagogical-environmental factors; ● psychosocial factors; ◆ medical factors; ▲ sleep-environmental factors. Doctors located 11 out of 52 ideas in the go-zone (upper right quadrant), of which most belonged to the Cluster pedagogical-environmental factors. The ideas in the go-zone had average ratings of ≥ 3.00 for both influence on sleep and occurrence in practice and were: 1) Screen use (TV, phone, tablet, computer) right before sleeping; 3) Worrying about something; 14) Exciting days; holidays, birthday; 17) Having no set bedtimes; 18) Going to bed too late/Being put to bed too late; 20) No set sleep/bedtime routine; 23) Having no clear day structure/rhythm; 47) Gaming before going to bed; 49) Parents who find it difficult to set limits; 51) Too much screen light (TV, phone, tablet, computer) in the evening (1-2 hours before going to bed); 52) Being ill/having a cold.

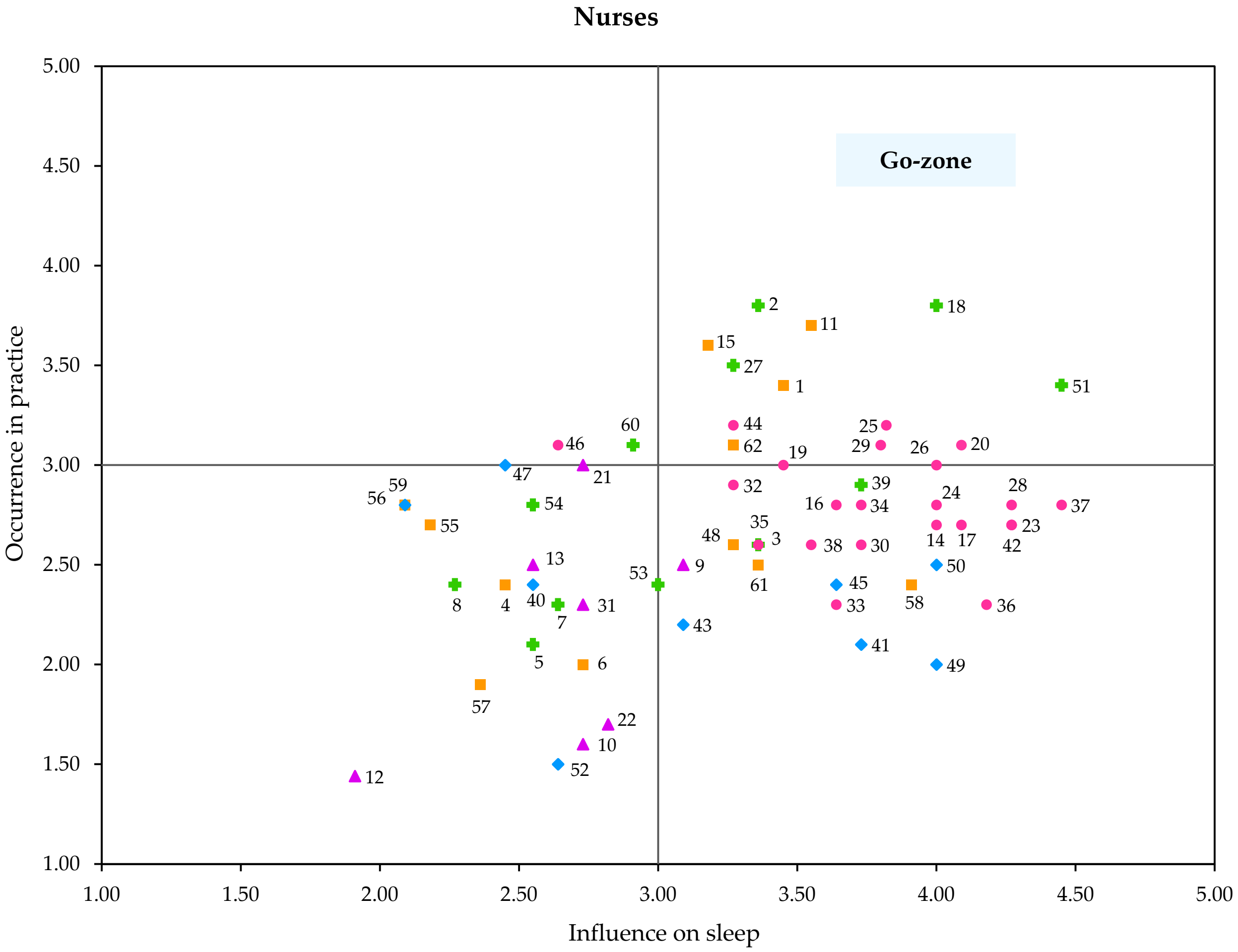

Figure A5. Go-zone plot nurses. Coloured shapes indicate to what cluster this idea belongs:

■ pedagogical-environmental factors; ✚ daytime and evening activities; ◆ medical factors; ● psychosocial factors; ▲ sleep-environmental factors. Nurses located 14 out of 62 ideas in the go-zone (upper right quadrant), of which most belonged to the Cluster psychosocial factors. The ideas in the go-zone had average ratings of ≥ 3.00 for both influence on sleep and occurrence in practice and were: 1) Having no rhythm and little structure in the daily routine; 2) Excessive use of computer or other screens (tablet, phone) during the day; 11) Going to bed too late/Being put to bed too late; 15) Having no set bedtimes; 18) Screen use (TV, phone, tablet, computer) just before going to sleep; 19) Being scared in a dark bedroom; 20) Stress due to arguing at home; 25) Not wanting to be alone; 26) Changes in daily life: moving, new school, holidays; 27) Having had inadequate amount of relaxation before going to bed, causing the brain to be too active; 29) A daytime event that still affects the child; 44) Having watched something exciting on TV or the internet; 51) Free use of screens (TV, phone, tablet, computer) in the bedroom; 62) Having no bedtime ritual or sleep-related rules and no consequences for following the rules.

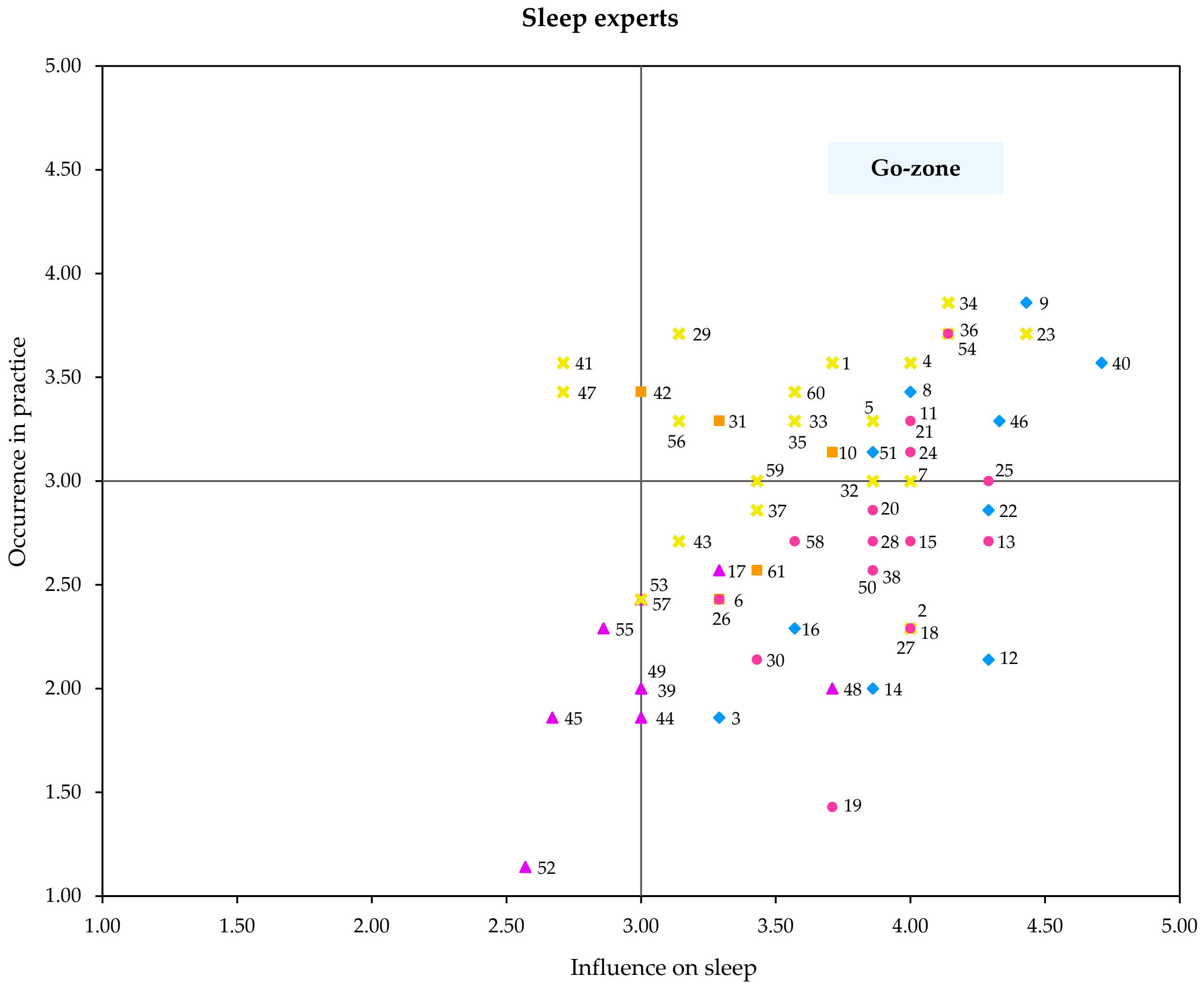

Figure A6. Go-zone plot sleep experts. Coloured shapes indicate to what cluster this idea belongs: ✖ sleep hygiene factors; ■ pedagogical-environmental factors; ◆ medical factors; ● psychosocial factors; ▲ sleep-environmental factors. Sleep experts located 27 out of 52 ideas in the go-zone (upper right quadrant), of which most belonged to the Cluster sleep hygiene factors. The ideas in the go-zone had average ratings of ≥ 3.00 for both influence on sleep and occurrence in practice and were: 1) The sleep duration is not adjusted to the needs of the child; 4) Having irregular sleep times; 5) Doing activities until late in the evening; 7) Going to bed at the wrong time/Being put to bed at the wrong time (too early, too late); 8) Mental problems; 9) Having a disturbed biological clock; 10) Refusing to listen and keep testing the limits when going to bed; 11) Worrying in the evening/at night about problems at home; 21) Worrying about the day that has been; 23) Gaming/on the game console until just before going to sleep; 24) Worrying about the next day; 25) Changes in the home situation: divorce, death of a loved one; 29) Excessive gaming/on the game console during the day; 31) Parents who do not set clear rules or who are too strict; 32) Being physically active/doing sports late in the evening; 33) The lack of a sleep ritual; 34) Screen use just before going to sleep; 35) Doing homework until late in the evening; 36) Not having reduced stimuli during the day, and therefore still too mentally active when going to bed; 40) A sleep disorder; 42) Parents who take their children into their bed; 46) Disturbed melatonin production; 51) Medication use with effects on sleep, taking medication at the wrong time; 54) Worrying in the evening/at night about problems at school; 56) Too little light and fresh air during the day due to being inside too much; 59) Eating/Drinking disrupting food/drinks (caffeine, sugar or fat) before going to sleep; 60) Too little exercise during the day.

Supplementary D

Table A2. Cluster compositions of original ideas and mean influence on sleep[1] and occurrence in practice[2] ratings of doctors (N = 9), including the assignment of original ideas to perceived determinants.

Clusters	Original ideas	Idea nr.[3]	Merged to perceived determinant[4]	Mean	
				In	Oc
**1. Pedagogical-environmental factors**				*3.5*	*3.0*
	Screen use (TV, phone, tablet, computer) right before sleeping	1	Screen use before bedtime	**4.0**	**4.0**
	A lack of exercise during the day	2	Inadequate daytime PA	2.9	3.2
	Having no set bedtimes	17	Inconsistent sleep schedule	**3.4**	**3.2**
	Going to bed too late/Being put to bed too late	18	Inconsistent sleep schedule	**3.9**	**3.3**
	Having slept during the day	19	Daytime nap	3.4	2.4
	No set sleep/bedtime routine	20	No bedtime routine	**3.4**	**3.3**
	Being too little outside during the day	21	Inadequate time outside at daytime	2.9	3.1
	Having no clear day structure/rhythm	23	Inadequate structure of the day	**3.9**	**3.1**
	Going to bed too early/Being put to bed too early	24	Inadequate bedtime	2.9	1.9
	Drinking stimulating (high sugar and/or caffeinated) drinks in the evening	42	Sleep-disrupting food or drinks	3.8	2.4
	Eating within an hour before going to sleep	43	Eating close to bedtime	2.8	2.3
	Doing sports/training late in the evening (< 1 hour before the desired bedtime)	44	Evening PA	3.2	2.0
	Gaming before going to bed	47	Playing stimulating games before bedtime	**3.9**	**3.8**
	Parents who find it difficult to set limits	49	Parents' inability to set boundaries	**3.4**	**3.3**
	Intentionally slowing down before going to bed	50	Bedtime procrastination	3.1	2.6
	Too much screen light (TV, phone, tablet, computer) in the evening (1-2 hours before going to bed)	51	Screen use before bedtime	**4.1**	**4.0**
**2. Psychosocial factors**				*3.7*	*2.6*
	Worrying about something	3	Worrying	**3.8**	**3.3**
	Fear of parents not coming back	4	Fear	3.6	1.9
	Having stress about school	6	Worrying	3.8	2.9
	Feeling like the room is not safe	7	Feeling unsafe	3.8	2.1
	Fear of being alone	8	Fear	3.3	2.6
	Fear of dying while sleeping	9	Fear	3.6	1.3
	Worrying about the day that has been	10	Worrying	3.7	2.8
	Fight between parents	11	Stressful family situation	4.1	2.4
	A traumatic event	12	Traumatic event	4.1	2.2
	Being scared of one's own thoughts and fantasies	13	Fear	3.6	2.4
	Exciting days; holidays, birthday	14	A change in daily life	**3.6**	**3.2**

	Troubled home situation due to family problems that cause tension, divorce parents	15	Stressful family situation	4.3	2.8
	Having to process the day	16	Unprocessed thoughts or feelings	3.3	2.8
	Fear of the dark	22	Fear	3.4	2.8
	Worrying about the next day	29	Worrying	3.4	2.8
	Nightmares	35	Nightmares	3.6	2.8
	Unstable home situation due to verbal and/or physical violence	45	Stressful family situation	4.3	2.3
**3. Medical factors**				*3.5*	*2.4*
	Physical complaints/pain	5	Physical complaint	4.3	2.6
	Medical condition	34	Medical complaint	3.6	2.8
	Bed-wetting	36	Sleep disorder	3.2	2.6
	Medication use with effects on sleep	37	Medication use with effects on sleep	3.6	2.0
	Mental condition	38	Mental problems	3.3	2.2
	Using melatonin (sleeping pills) wrongly	40	Medication use with effects on sleep	3.7	2.1
	Heredity for sleep problems / hereditary	41	Heredity for sleep problems	2.8	1.8
	Being ill/having a cold	52	Illness	**3.1**	**3.0**
	Overweight/obesity	53	Medical complaint	2.4	2.7
	Sleep disorder	54	Sleep disorder	4.6	2.0
**4. Sleep-environmental factors**				*2.8*	*2.4*
	Bedroom too hot or too cold	25	Not the right temperature	3.0	2.0
	Sleeping with multiple people in a room	26	Bedroom sharing	2.8	3.0
	Too much ambient noise	27	Noise outside	3.1	2.1
	Room not dark enough	28	Too light	2.4	1.9
	Room not ventilated	30	Not ventilated	2.7	1.9
	Not having a bedroom or sleeping in another place in the house	31	No bedroom	3.1	2.7
	Sleeping in bed with parents or other family member	32	Co-sleeping	3.0	2.8
	Light from outside the bedroom	33	Too light	2.7	1.8
	Sleeping with multiple children in one bedroom	39	Bedroom sharing	2.7	3.2

N = Number of participants; In = Influence on sleep; Oc = Occurrence in practice; PA = Physical activity. [1] The average influence on sleep rating is based on the question: 'How much does this [idea] negatively influence sleep?' answered on a 5-point Likert scale from 'no influence at all = 1' to 'great influence = 5'; [2] The average occurrence in practice rating is based on the question: 'How often does this [idea] occur in practice?' answered on a 5-point Likert scale from 'never = 1' to 'always = 5'; [3] Idea nr. = The number of the generated idea in this group. This corresponds with the numbers on the concept maps in Appendix B and go-zone plots in Appendix C; [4] Merged into perceived determinant = The overall perceived determinant to which the original idea of this group is assigned. Bold values indicate ideas rates as important (with influence on sleep and occurrence in practice ratings ≥ 3.00); Cursive values indicate the overall mean rating per cluster.

Table A3. Cluster compositions of original ideas and mean influence on sleep[1] and occurrence in practice[2] ratings of nurses (N = 11), including the assignment of original ideas to perceived determinants.

Clusters	Original ideas	Idea nr.[3]	Merged to perceived determinants[4]	Mean[5]	
				In	Oc
**1. Pedagogical-environmental factors**				*2.9*	*2.8*
	Having no rhythm and little structure in the daily routine	1	Inadequate structure of the day	**3.5**	**3.4**
	Secretly reading a book in bed[5]	4	Bedtime procrastination	2.5	2.4
	Being hungry or thirsty	6	Inadequate amount of food or drink	2.7	2.0
	Going to bed too late/Being put to bed too late	11	Inconsistent sleep schedule	**3.6**	**3.7**
	Having no set bedtimes	15	Inconsistent sleep schedule	**3.2**	**3.6**
	Refusing to go to bed[5]	48	Bedtime resistance	3.3	2.6
	Comparing oneself to classmates who go to bed later[5]	55	Social norm for bedtime at school	2.2	2.7
	Sleeping in the parents' bed	56	Co-sleeping	2.1	2.8
	Children demand a too elaborate bedtime ritual before going to sleep	57	Bedtime procrastination	2.4	1.9
	Having slept during the day	58	Daytime nap	3.9	2.4
	Uncertainty among parents about what an appropriate bedtime is	59	Insufficient parental sleep-related knowledge	2.1	2.8
	Trying to get attention for not wanting to go to sleep	61	Parents' negative focus on sleep	3.4	2.5
	Having no bedtime ritual or sleep-related rules and no consequences for following the rules	62	No bedtime routine	**3.3**	**3.1**
**2. Daytime and evening activities**				3.2	2.9
	Excessive use of computer or other screens (tablet, phone) during the day	2	Excessive daytime screen use	**3.4**	**3.8**
	Playing exciting games before going to sleep	3	Playing stimulating games before bedtime	3.4	2.6
	Playing outside until late	5	Evening PA	2.6	2.1
	Doing sports too late in the evening	7	Evening PA	2.6	2.3
	Eating just before going to sleep	8	Eating close to bedtime	2.3	2.4
	Screen use (TV, phone, tablet, computer) just before going to sleep	18	Screen use before bedtime	**4.0**	**3.8**
	Having had inadequate amount of relaxation before going to bed, causing the brain to be too active	27	Inadequate time to relax	**3.3**	**3.5**
	Watching a movie until late before going to sleep	39	Screen use before bedtime	3.7	2.9
	Free use of screens (TV, phone, tablet, computer) in the bedroom	51	Screens in the bedroom	**4.5**	**3.4**
	Having done too many daytime activities	53	Too many daytime activities	3.0	2.4
	Inadequate amount of playing outside and too much of playing inside	54	Inadequate time outside	2.6	2.8

	A lack of physical exercise during the day	60	Inadequate daytime PA	2.9	3.1
**3. Medical factors**				*3.3*	*2.3*
	Bed-wetting	40	Sleep disorder	2.6	2.4
	Being ill	41	Illness	3.7	2.1
	Medical complaints	43	Medical complaint	3.1	2.2
	Mental complaints	45	Mental problems	3.6	2.4
	Overweight/obesity	47	Medical complaint	2.5	3.0
	Medication use with effects on sleep	49	Medication use with effects on sleep	4.0	2.0
	Having a disturbed biological clock[7]	50	Disturbed biological clock	4.0	2.5
	Sleep walking	52	Sleep disorder	2.6	1.5
**4. Psychosocial factors**				*3.8*	*2.8*
	Being scared of one's own thoughts, fantasies or stories that go around	14	Fear	4.0	2.7
	Feeling stressed about something that will happen the next day	16	Upcoming stressful event	3.6	2.8
	Worrying about family members or friends	17	Worrying	4.1	2.7
	Being scared in a dark bedroom	19	Fear	**3.5**	**3.0**
	Experiencing stress due to arguing at home	20	Stressful family situation	**4.1**	**3.1**
	Experiencing stress due to arguing with someone	23	Worrying	4.3	2.7
	Worrying about school: a test, disappointing results, not being able to keep up in school, being scared of not doing well	24	Worrying	4.0	2.8
	Not wanting to be alone	25	Fear	**3.8**	**3.2**
	Changes in daily life: moving, new school, holidays	26	A change in daily life	**4.0**	**3.0**
	Being bullied	28	Being bullied	4.3	2.8
	A daytime event that still affects the child	29	Worrying	**3.8**	**3.1**
	Feeling pressure to perform well	30	Performance pressure	3.7	2.6
	Setting high expectations for themselves	32	Performance pressure	3.3	2.9
	Missing a parent or waiting for a parent to come home	33	Loneliness	3.6	2.3
	Experiencing unprocessed emotions of that day	34	Unprocessed thoughts or feelings	3.7	2.8
	Feeling lonely	35	Loneliness	3.4	2.6
	Not feeling safe at home or at school	36	Feeling unsafe	4.2	2.3
	Parents who are in a divorce	37	Stressful family situation	4.5	2.8
	Scary dreams/nightmares	38	Nightmares	3.6	2.6
	Having had a traumatic experience that comes back in bed	42	Traumatic event	4.3	2.7
	Having watched something exciting on TV or the internet	44	Unprocessed thoughts or feelings	**3.3**	**3.2**
	The child's temperament[7]	46	Child's temperament	2.6	3.1

**5. Sleep-environmental factors**				*2.7*	2.2
	Noise in the neighbourhood/outside	9	Noise outside	3.1	2.5
	Too much light in the bedroom	10	Too much light	2.7	1.6
	Too much clutter or stuffed pets in the room	12	Untidy bedroom	1.9	1.4
	Living in a small house with a lot of people	13	Cramped housing	2.6	2.5
	Sleeping in a room with (a) brother(s) and/or sister(s)	21	Bedroom sharing	2.7	3.0
	Too cold or too warm in the bedroom	22	Not the right temperature	2.8	1.7
	Noise in the house	31	Noise inside	2.7	2.3

N = Number of participants; In = Influence on sleep; Oc = Occurrence in practice; PA = Physical activity. [1] The average influence on sleep rating is based on the question: 'How much does this [idea] negatively influence sleep?' answered on a 5-point Likert scale from 'no influence at all = 1' to 'great influence = 5'; [2] The average occurrence in practice rating is based on the question: 'How often does this [idea] occur in practice?' answered on a 5-point Likert scale from 'never = 1' to 'always = 5'; [3] Idea nr. = The number of the generated idea in this group. This corresponds with the numbers on the concept maps in Appendix B and go-zone plots in Appendix C; [4] Merged into perceived determinant = The overall perceived determinant to which the original idea of this group is assigned; [5] this idea was moved from Cluster 5 to Cluster 1; [6] this idea was moved from Cluster 2 to Cluster 3; [7] this idea was moved to Cluster 4. Bold values indicate ideas rates as important (with influence on sleep and occurrence in practice ratings ≥ 3.00); Cursive values indicate the overall mean rating per cluster.

Table A4. Cluster compositions of original ideas and mean influence on sleep[1] and occurrence in practice[2] ratings of sleep experts (N = 7), including the assignment of original ideas to perceived determinants.

Clusters	Original ideas	Idea nr.[3]	Merged to perceived determinant[4]	Mean[5]	
				**In**	**Oc**
**1. Sleep hygiene factors**				*3.6*	*3.3*
	The sleep duration is not adjusted to the needs of the child	1	Inadequate bedtime	**3.7**	**3.6**
	Sleeping (too much) during the day	2	Daytime napping	4.0	2.3
	Having irregular sleep times	4	Inconsistent sleep schedule	**4.0**	**3.6**
	Doing activities until late in the evening	5	Inadequate time to relax	**3.9**	**3.3**
	Going to bed at the wrong time/Being put to bed at the wrong time (too early, too late)	7	Inadequate bedtime	**4.0**	**3.0**
	Gaming/on the game console until just before going to sleep	23	Playing stimulating games before bedtime	**4.4**	**3.7**
	Excessive gaming/on the game console during the day	29	Excessive daytime screen use	**3.1**	**3.7**
	Being physically active/doing sports late in the evening	32	Evening PA	**3.9**	**3.0**
	The lack of a sleep ritual	33	No bedtime routine	**3.6**	**3.3**
	Screen use just before going to sleep	34	Screen use before bedtime	**4.1**	**3.9**
	Doing homework just until late in the evening	35	Inadequate time to relax	**3.6**	**3.3**

	Not having reduced stimuli during the day, and therefore still too mentally active when going to bed	36	Inadequate time to relax	**4.1**	**3.7**
	Too much light in the evening	37	Too much evening light	3.4	2.9
	Screen use during the day	41	Excessive daytime screen use	2.7	3.6
	Too little light in the morning	43	Inadequate morning light	3.1	2.7
	Using the bed for more than just sleeping, playing in bed, watching TV in bed	47	Bed also used for other activities	2.7	3.4
	Too little light and fresh air during the day due to being inside too much	56	Inadequate time outside at daytime	**3.1**	**3.3**
	Eating/drinking too late	57	Eating/drinking close to bedtime	3.0	2.4
	Eating/Drinking disrupting food/drinks (caffeine, sugar, or fat) before going to sleep	59	Sleep-disrupting food or drinks	**3.4**	**3.0**
	Too little exercise during the day	60	Inadequate daytime PA	**3.6**	**3.4**
**2. Pedagogical-environmental factors**				*3.3*	*2.9*
	(Separated) parents applying different rules regarding bed times	6	Different bedtime rules between parents	3.3	2.4
	Refusing to listen and continuing to test the limits when going to bed[5]	10	Bedtime procrastination	**3.7**	**3.1**
	Parents who do not set clear rules or who are too strict[5]	31	Lack of clear rules	**3.3**	**3.3**
	Parents who take their children into their bed	42	Co-sleeping	**3.0**	**3.4**
	Parents who create negative attention around sleep by putting too much emphasis on having to sleep	61	Parents' negative focus on sleep	3.4	2.6
**3. Medical factors**				*4.1*	*2.8*
	Cold feet	3	Physical complaint	3.3	1.9
	Mental problems[6]	8	Mental problems	**4.0**	**3.4**
	Having a disturbed biological clock	9	Disturbed biological clock	**4.4**	**3.9**
	Being stuffy	12	Medical complaint	4.3	2.1
	Growing pain in the legs	14	Physical complaint	3.9	2.0
	Allergy/itch	16	Medical complaint	3.6	2.3
	Illness/pain	22	Illness	4.3	2.9
	Sleep disorder	40	Sleep disorder	**4.7**	**3.6**
	Disturbed melatonin production	46	Sleep disorder	**4.3**	**3.3**
	Medication use with effects on sleep, taking medication at the wrong time[7]	51	Medication use with effects on sleep	**3.9**	**3.1**
**4. Psychosocial factors**				*3.9*	*2.7*
	Worrying in the evening/at night about problems at home	11	Worrying	**4.0**	**3.3**
	Being bullied and worrying about this in the evening/at night	13	Being bullied	4.3	2.7
	Problems between parent(s) and child	15	Stressful family situation	4.0	2.7
	Stress and agitation of the parent that passes on to the child	17	Parental stress	3.3	2.6
	The bedroom and bed are not seen as safe	18	Feeling unsafe	4.0	2.3

	Being scared of the unsafe and troubled neighbourhood they live in	19	Fear	3.7	1.4
	Not feeling like going to sleep	20	Bedtime resistance	3.9	2.9
	Worrying about the day that has been	21	Worrying	**4.0**	**3.3**
	Worrying about the next day	24	Worrying	**4.0**	**3.1**
	Changes in the home situation; divorce, death of a loved one	25	A change in daily life	**4.3**	**3.0**
	Experiencing fear of nightmares	26	Fear	3.3	2.4
	Experiencing stress due to arguing at home	27	Fear	4.0	2.3
	Scared of the dark	28	Fear	3.9	2.7
	Scared of parent(s) leaving	30	Fear	3.4	2.1
	A traumatic experience from the past	38	Traumatic event	3.9	2.6
	Being scared of own ideas and fantasies	50	Fear	3.9	2.6
	Worrying in the evening/at night about problems at school	54	Worrying	**4.1**	**3.7**
	Scary or bad dreams that wake a child	58	Nightmares	3.6	2.7
**5. Sleep-environmental factors**				*2.9*	*2.0*
	Ambient noise at home	39	Noise inside	3.0	2.0
	Noise outside	44	Noise outside	3.0	1.9
	Bedroom too hot or too cold	45	Not the right temperature	2.7	1.9
	Sleeping in a bedroom with someone who keeps the child awake	48	Bedroom sharing	3.7	2.0
	Uncomfortable bed	49	Uncomfortable sleeping place	3.0	2.0
	Uncomfortable sleep wear	52	Uncomfortable sleep wear	2.6	1.1
	Not dark enough in the bedroom	53	Too much light	3.0	2.4
	Untidy bedroom	55	Untidy bedroom	2.9	2.3

N = Number of participants; In = Influence on sleep; Oc = Occurrence in practice; PA = Physical activity. [1] The average influence on sleep rating is based on the question: 'How much does this [idea] negatively influence sleep?' answered on a 5-point Likert scale from 'no influence at all = 1' to 'great influence = 5'; [2] The average occurrence in practice rating is based on the question: 'How often does this [idea] occur in practice?' answered on a 5-point Likert scale from 'never = 1' to 'always = 5'; [3] Idea nr. = The number of the generated idea in this group. This corresponds with the numbers on the concept maps in Appendix B and go-zone plots in Appendix C; [4] Merged into perceived determinant = The overall perceived determinant to which the original idea of this group is assigned; [5] this idea was moved from Cluster 4 to Cluster 2; [6] this idea was moved from Cluster 4 to Cluster 3; [7] this idea was moved from Cluster 5 to Cluster 3. Bold values indicate ideas rates as important (with influence on sleep and occurrence in practice ratings ≥ 3.00); Cursive values indicate the overall mean rating per cluster.

Supplementary E

Table A5. Results of validation study (N=16), including conceptual fit, missing determinants, and improvement of name per category.

**Categories**	**Category's conceptual fit**				**Missing determinants in category**			**Improvement of category's name**	
	Mean[1]	***Determinants perceived as less appropriate in category (mentioned by nr. of participants)***	***Suggested category (mentioned by nr. of participants)***	***Changed to category***	***Determinants perceived as missing from category (mentioned by nr. of participants)***	***Perceived determinant from original study***	***New perceived determinant***	***Suggestions for improving the name of category***	***New name***
**Pedagogical determinants**	4.2	Bedtime resistance (n=3)	Child determinants (n=1); Psychosocial determinants (n=1); Behavioural determinants (n=1)	Psychosocial determinants	No time to put child to bed (n=1)		Having no time to put child to bed	Relevant parenting determinants	
		Social norm for bedtime at school (n=2)	Environmental determinants (n=1); Societal determinants (n=1)		Rules for screen use before bedtime (n=1)	Lack of clear rules		Subgroups: parenting, parents' knowledge, nutrition, parenting style, parents' behaviour	
		Inadequate amount of food or drink (n=2)	Environmental determinants (n=1); Behavioural determinants (n=1)		Parents set the wrong example (n=2)		Parents set the wrong example		
		Co-sleeping (n=1)	Sleep-environmental determinants (n=1)		Rules for playing outside (n=1)	Lack of clear rules			
					Social norm for playing outside (n=1)		Social norm for playing outside		
					Responsivity (knowledge, ability, and skills to respond adequately to night terrors, trouble falling asleep, and		Parents' ability to respond to the child's sleep problems		

					sleeping through the night (n=1)				
					Cramped housing	Cramped housing			
					Sharing a bedroom with children of varying ages/bedtimes	Bedroom sharing			
					Cultural influence (parents that learned to sleep differently than in Europe); sharing a bedroom with multiple people or siblings (n=1)	Bedroom sharing			
					Parental stress (n=1)	Parental stress			
					Difference between siblings (n=1)	Bedroom sharing			
					Noise in the house (n=1)	Noise inside			
					Too busy schedule during the day (n=1)	Too many daytime activities			
**Behavioural determinants**	3.8	Eating/drinking close to bedtime (n=1)	Pedagogical determinants (n=1)		TV always turned on; overstimulation (n=1)	Excessive daytime screen use; Screen use before bedtime		Parental influence on lifestyle	Daytime and evening activities
		Inadequate bedtime (n=6)	Pedagogical determinants (n=6)	Pedagogical determinants	Sleep-disrupting food; sugary drinks (n=1)	Sleep-disrupting food or drinks		Pedagogical determinants and lack of parents' knowledge	
		Sleep-disrupting food or drinks (n=1)	Environmental determinants (n=1)		Parental tiredness (n=1)	Parental stress		Subgroups: screens, nutrition, activities	
		Inadequate morning light (n=3)	Environmental determinants (n=2)		Too busy playing around just before going to bed (n=1)	Playing stimulating games before bedtime			

Too much evening light (n=3)	Environmental determinants (n=2)	Using the bed as punishment (n=1)	Parents' negative focus on sleep
Too many daytime activities (n=2)	Pedagogical determinants (n=1)		
Screens in the bedroom (n=3)	Pedagogical determinants (n=1); Sleep-environmental determinants (n=2)	Putting too much focus on having to go to bed (n=1)	Parents' negative focus on sleep
Screen use before bedtime (n=2)	Pedagogical determinants (n=2)		
Inadequate time to relax (n=1)	Pedagogical determinants (n=1)	Overstimulation during the day (n=1)	Unprocessed thoughts or feelings; Too many daytime activities
Some behavioural determinants (n=3)	Pedagogical determinants (n=1); Psychosocial determinants (n=1); Environmental determinants (n=1)	Lack of exercise during the day (n=1)	Inadequate daytime PA
		Not taking time to relax before bedtime (n=1)	Inadequate time to relax
		Refusing to go to sleep (n=1)	Bedtime procrastination
		Being active late at night (n=1)	Evening PA
		Doing homework (n=1)	Inadequate time to relax
		Imbalance exertion/relaxation (n=1)	Inadequate time to relax; Too many daytime activities

**Psychosocial determinants**	4.3	Parental stress (n=1)	Pedagogical determinants (n=1)	Quality of parents' relationship (n=1)	Stressful family situation;<br>Parental stress		Psychosocial child determinants
		Nightmares (n=1)		Negative self-image (n=1)		Negative self-image	
				Worrying (n=1)	Worrying		
				High sensitivity/stimuli (n=1)		High sensitivity	
				School results (n=1)	Worrying;<br>Unprocessed thoughts or feelings		
				Parents' thoughts on sleep (n=1)		Parents' attitude towards sleep	
				Parents' environment (opinions of others, small support system, daily structure/meaningful daily activities, mental health, patience) (n=1)		Parents' resilience	
				Need for undivided attention from parent(s)/searching for connection (n=1)		A lack of attention from parent(s)	
				Processing stimuli at school (n=1)	Worrying;<br>Unprocessed thoughts or feelings		
				Stress about school (n=1)	Worrying;<br>Performance pressure;<br>Unprocessed thoughts or feelings		
**Medical determinants**	4.1	Mental problems (n=1)	Psychosocial determinants (n=1)	ADHD (n=1)	Mental problems		Physical or genetic determinants
				High sensitivity (n=2)		High sensitivity	(Bio)medical determinants

				Allergies (n=1)	Medical complaint		
				Nutrition/drinking problems (n=1)	Inadequate amount of food or drink; Sleep-disrupting food or drinks; Eating/drinking close to bedtime; Medical complaint		
				Pain (n=2)	Illness; Physical complaint		
				Processing stimuli (n=2)	Worrying; Unprocessed thoughts or feelings		
**Sleep-environmental determinants**	4.1	Untidy bedroom (n=1)	Pedagogical determinants (n=1)	No suitable bed (n=1)	Uncomfortable sleeping place		Physical sleep-environmental determinants
				Sharing a bed (n=1)	Co-sleeping		
				Screens in the bedroom (n=2)	Screens in the bedroom		
				Too many stuffed toys etc. in the bed (n=1)	Untidy bedroom		
				Too dark (n=2)	Fear (of the dark)		
				Uncomfortable sleep wear/materials (n=1)	Uncomfortable sleeping place; Uncomfortable sleep wear		
				Feeling unsafe due to absence of stuffed toy (n=1)		Absence of stuffed toy	
				Be cold or warm (n=1)	Physical complaint		
				Noise in the house (n=1)	Noise inside		

	Feeling unsafe due to large house (n=1)	Feeling unsafe

[1] Mean = mean conceptual fit rating based on the question: 'To what extent do you think the factors in the group fit together' answered on a 5-point Likert scale from 'not well at all = 1' to 'very well = 5'.
